# Micro- and Macroscale Patterns of Petal Morphogenesis in *Nigella damascena* (Ranunculaceae) Revealed by Geometric Morphometrics and Cellular Analyses

**DOI:** 10.3389/fpls.2021.769246

**Published:** 2021-11-19

**Authors:** Pierre Galipot, Sylvain Gerber, Martine Le Guilloux, Florian Jabbour, Catherine Damerval

**Affiliations:** ^1^Institut de Systématique, Evolution, Biodiversité (ISYEB), Muséum National d’Histoire Naturelle, CNRS, Sorbonne Université, EPHE, Université des Antilles, Paris, France; ^2^Génétique Quantitative et Evolution-Le Moulon, Université Paris-Saclay, INRAE, CNRS, AgroParisTech, Gif-sur-Yvette, France

**Keywords:** *Nigella damascena*, petal, allometry, geometric morphometrics, morphogenesis, Ranunculaceae, development

## Abstract

Petals, the inner organs in a differentiated perianth, generally play an important role in pollinator attraction. As such they exhibit an extraordinary diversity of shapes, sizes, and colors. Being involved in pollinator attraction and reward, they are privileged targets of evolution. The corolla of the Ranunculaceae species *Nigella damascena* consists of elaborate nectariferous petals, made of a stalk, upper, and lower lips forming a nectar pouch, shiny pseudonectaries, and pilose ears. While the main events of petal development are properly described, a few is known about the pattern of organ size and shape covariation and the cellular dynamics during development. In this study, we investigated the relationships between morphogenesis and growth of *N. damascena* petals using geometric morphometrics coupled with the study of cell characteristics. First, we found that petal shape and size dynamics are allometric during development and that their covariation suggests that petal shape change dynamics are exponentially slower than growth. We then found that cell proliferation is the major driver of shape patterning during development, while petal size dynamics are mostly driven by cell expansion. Our analyses provide a quantitative basis to characterize the relationships between shape, size, and cell characteristics during the development of an elaborate floral structure. Such studies lay the ground for future evo-devo investigations of the large morphological diversity observed in nectariferous structures, in Ranunculaceae and beyond.

## Introduction

In the flowers of most angiosperms, sterile structures, collectively called the perianth, surround fertile organs and are organized in two functionally and morphologically differentiated organ types. While the most external organs, i.e., the sepals, play a protective role for the developing stamens and carpels, the innermost perianth organs, i.e., the petals, generally carry an attractive role for pollinators, based on a particular shape, size, color, and sometimes fragrance. Elaborate petals, as opposed to simple petals, have been described in various eudicot groups. They have evolved a variety of patterns, such as lobes, spurs, and specialized trichomes (Endress and Matthews, 2006).

In the early-diverging eudicot family Ranunculaceae, a large diversity of petal forms is found, from simple laminas with a tiny nectariferous scale, as in *Ranunculus*, to highly elaborate nectariferous spurred organs, as in *Aquilegia*. Floral development and, to a lesser extent, petal development have already been described qualitatively using the classical microscopy techniques (Erbar et al., 1999; Tucker and Hodges, 2005; Jabbour et al., 2009, 2015; Ren et al., 2009; Zhao et al., 2011). A detailed investigation of the development of the elaborate petal has been conducted in the genus *Nigella* using histology and micro-CT (Yao et al., 2019). It has been shown that petal complexity is progressively acquired during development, as different elaborated features appear sequentially, giving rise to a mature petal that comprises a stalk, upper, and lower lips, a nectar pouch, lobes, pseudonectaries (Liao et al., 2020), trichomes, and color patterns. Although some of these features are present in all *Nigella* species, others are shared by few species only (Yao et al., 2019). Nevertheless, the precise quantification of petal shape transformation, as well as the covariation of petal shape with petal growth, during development, is still lacking.

From a developmental perspective, petals are lateral organs initiated as groups of cells on the flank of the floral meristem. After polarity establishment, the transitions from the period of cell proliferation to expansion and differentiation in space and time are key to shape acquisition. The shape is altered during development due to variation in local growth rates and anisotropy (for review, Whitewoods and Coen, 2017). In model species such as *Arabidopsis thaliana* and *Antirrhinum majus*, clonal cell analysis has provided information on the growth processes at play, opening the way to mathematical modeling (Rolland-Lagan et al., 2003; Sauret-Güeto et al., 2013). When the study species is not amenable to such cell lineage marking, the analyses of cell proliferation, cell counting, and measurements can give clues about the processes directing shape variation, as demonstrated in the spurred petals of *Aquilegia*, *Centranthus*, and *Linaria* (Puzey et al., 2012; Mack and Davis, 2015; Cullen et al., 2018). Still, at a more integrated level, the quantification of allometry during development with the geometric morphometric methods provides invaluable information on form transformation (Gerber, 2014; Klingenberg, 2016).

**FIGURE 1:**
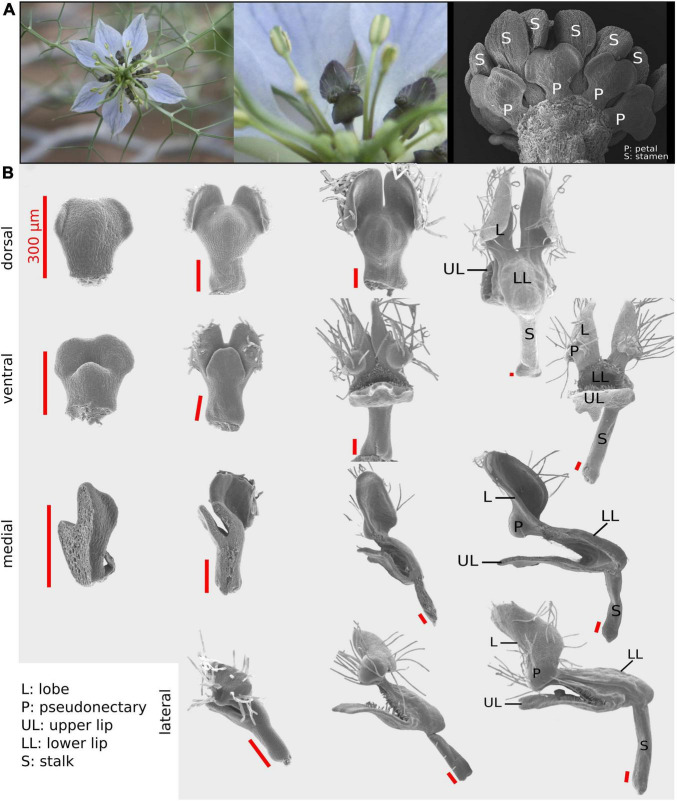
The *Nigella damascena* petal and its developmental sequence. **(A)** Wild-type flowers of *N. damascena* comprise 7–9 petals. **(B)** Four views have been used to recapitulate petal morphogenesis: dorsal, ventral, medial, and lateral. On every column, petals are approximately at the same developmental stage. First column petals are taken after the restart of petal development (which undergoes a stasis after petal primordia formation). In the last column, petal morphogenesis is almost complete and all elaborations are present.

As in other *Nigella* species, the mature petal of *Nigella damascena* is composed of several differentiated domains (Figure 1). These domains differentiate progressively during development from a simple lamina (Jabbour et al., 2015). Their respective contribution to the overall petal shape and size during development is still to be described. Shape and size are two characteristics that may theoretically change independently during development. While shape and size are often treated conceptually and analytically as distinct components of form, their dynamics are, in fact, often governed by common processes (e.g., cell proliferation or cell expansion). Nevertheless, other processes seem to preferentially affect shape (e.g., cell rearrangements) or size (e.g., homogeneous cell expansion). For these reasons, there is no universal rule governing shape and size dynamics during development (Klingenberg, 2016).

In this study, we conducted a geometric morphometric study coupled to cellular investigations to assess the pattern of shape and size variation and covariation during the development of *N. damascena* petal. We found that covariation between shape and size is linearized when considering the log of size, suggesting an exponential allometric relationship between these variables. By repeating the study with different domains of the petal (stalk and nectar pouch) with distinct geometries, we found that this relationship seems to hold at these subscales, suggesting that this exponential relationship is based on general mechanisms. As cell expansion and cell division are the two main processes that influence petal size and shape, we followed their dynamics during petal development through the cell map analysis. We then managed to measure their relative contributions to petal size and shape changes. Focusing on the petal dorsal view, corresponding to the lower lip of the nectar pouch, we found that cell proliferation is prevalent in the first stages, which correspond to the period when most of the mature shapes are achieved. We then found that, in late stages, cell expansion takes over and controls the final growth of the petal but without leading to major shape changes.

## Materials and Methods

### Scanning Electron Microscopy

A total of 121 terminal flower buds were sampled from *N. damascena* plants grown in a growth chamber or in a greenhouse, from 8 days after floral transition to preanthetic flowers, for a time period of about 3 weeks. The sampling was designed to cover the time period of petal development and shape acquisition after the lag time following petal initiation (Jabbour et al., 2015). Floral buds were fixed in FAA (90% ethanol 70%, 5% formalin, 5% acetic acid) and then dissected under a stereoscope (Nikon, Tokyo, Japan SMZ 745T). Petals were then dehydrated in an ethanol series (70%, 80%, absolute alcohol) and dried using an Emitech K850 critical-point dryer (Quorum Technologies, Laughton, United Kingdom). Samples were mounted on the aluminum stubs with colloidal graphite, sputter-coated with platinum using an EM ACE600 fine coater (Leica, Wetzlar, Germany), and observed using a SU3500 scanning electron microscope (SEM, Hitachi, Tokyo, Japan).

Four different views (i.e., dorsal, ventral, medial-sagittal, and lateral-sagittal; Figure 1) were used to satisfactorily capture the three-dimensional (3D) form of *N. damascena* petal throughout development. Among the 121 dissected petals, 32 were mounted for dorsal view observation, 33 for ventral view, 36 for sagittal section observed from the medial side (hereafter “medial”), and 20 for sagittal section observed from the lateral side (hereafter “lateral”) (Figure 1). The medial and lateral views were informative regarding the shape of both the nectar pouch and the lobes.

### Geometric Morphometrics

Both shape and size of the growing petal were characterized morphometrically from the 2D configurations of landmarks defined for each of the four views. These configurations were chosen to ensure that the landmarks could be unambiguously identified and digitized on all specimens across the entire developmental phase studied. Consequently, some structures that were not yet present at the early stages, such as pseudonectaries, had to be ignored from our morphometric scheme. We also chose not to consider the lobed structures (except for their radial ridge), since they start to fold precociously in development, thereby precluding the definition of reliable landmarks.

**FIGURE 2:**
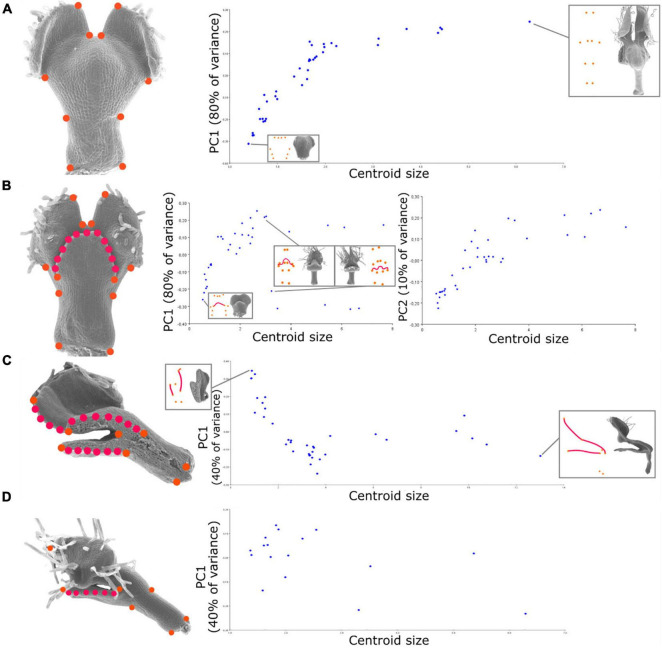
Relationship between shape and size during petal development revealed by geometric morphometrics of the developing *N. damascena* petal in the four views (i.e., dorsal, ventral, medial, and lateral). On the left side of **(A–D)** panels, landmark sets are indicated and superimposed to the corresponding petal scanning electron microscopy (SEM) pictures (orange dots are landmarks, and red dots are semi-landmarks). For the four views, the Procrustes analyses are followed by the principal component analysis (PCA), and its first axis (and also the second axis for ventral view) is plotted against centroid size (proxy of petal size). The first axis explains 80% of the variance for dorsal and ventral views and 40% of the variance for medial and lateral views. The percentage of variance explained by an axis corresponds to the ratio between the variance of that principal component and the total variance.

We used 12, 10, 8, and 7 landmarks (for ventral, dorsal, medial, and lateral, respectively) and 1, 3, and 1 series of 25 semi-landmarks (for ventral, medial side, and lateral side, respectively) to capture the homologous curved outlines of the lower lip (ventral and sagittal sides) and the petal lobes (sagittal sides) (Figure 2).

Landmark coordinates were manually recorded using tpsDig2 (Rohlf, 2015) and processed following standard procedures with tpsRelw (Rohlf, 2015). Landmark configurations were optimally aligned by generalized Procrustes analysis (Dryden and Mardia, 2016), which extract shape data by filtering out differences in scale, location, and orientation among raw landmark configurations. The minimum bending energy was used as a criterion to slide the semi-landmarks. The statistical analyses of shape variation were then carried out from the shape tangent coordinates. Notably, the landmark configurations for the ventral and dorsal views are the instances of configurations exhibiting bilateral object symmetry. Landmarks have been digitized on both sides, but only the symmetric component of shape variation was considered in subsequent analyses by averaging each specimen with its reflected copy. The patterns of shape variation were explored using the principal component analysis (PCA) (Jolliffe, 1986), and the patterns of ontogenetic allometry were investigated with multivariate regression of shape on size (i.e., centroid size).

**FIGURE 3:**
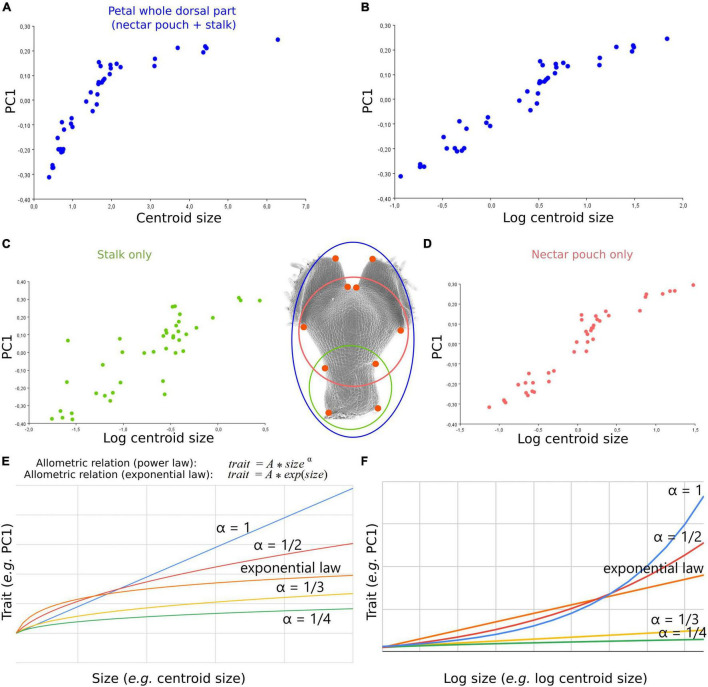
An allometric relation, potentially exponential, links shape and size dynamics of *N. damascena* whole petal from dorsal view and its subdomains (stalk and nectar pouch) during morphogenesis. **(A)** The first axis of PCA of dorsal view plotted against centroid size (proxy of petal size) and log of centroid size, and **(B)** shows that their covariation is well approximated by an exponential law. The same analysis was performed on the subsets of landmarks delimiting stalk only **(C)** and nectar pouch only **(D)**. Comparison between exponential and power-law allometric relations of a trait (PC1 in this study) and size **(E)** or log size **(F)**.

We applied the same protocol on the subsets of landmarks corresponding to petal subdomains. Both stalk and nectar pouch were the only domains of the petal that could be identified at every stage of development. They were separately analyzed to examine their particular growth dynamics and possible contributions to the overall shape changes (Figures 3C,D).

### Cell Characteristics in the Nectar Pouch Domain

We investigated cell characteristics in the nectar pouch domain using the area delimited by six landmarks in the dorsal view. We took advantage of the fact that cell boundaries are visible on SEM images to (i) count cells and measure the total surface of the domain, making it possible to calculate the mean cell surface for each sample; (ii) transform the nectar pouch domain into a tessellating network using Fiji software (Schindelin et al., 2012), in order to visualize cell populations that may differ by shape, size, and number during development. All the SEM pictures where cell boundaries were clearly visible have been used, which corresponds to 17 petals, covering the same developmental sequence as in the geometric morphometric analyses.

Cell area and elongation analyses and color representations were performed with Fiji MorphoLibJ plugins (Legland et al., 2016). Violin plots were produced with R (version 4.0.5) and ggplot2 library (version 3.3.2).

We used a unique cell color coding for all petals in order to be able to visually compare the stages (i.e., all cell area and cell elongation maps are available in Supplementary Figure 1). We then extracted cell area and elongation distribution using violin plots, the latter being plotted against the total cell number for each specimen.

Since the petals are not planar objects, the deviations from a perfect plane model were estimated by angle measurements on medial and lateral views in Fiji software, and they were shown to be an order of magnitude smaller than the apparent size differences among cells. This implies that the observed differences cannot be explained only by an effect of focal length distortion and angle of view effect.

### Relative Contributions of Cell Proliferation and Cell Expansion to Shape Transformation

**FIGURE 4:**
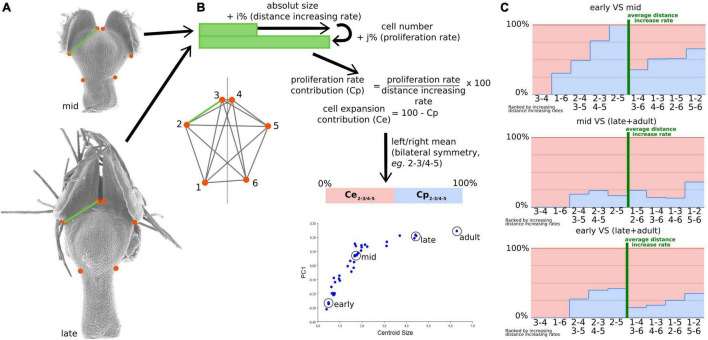
The relative contribution of cell proliferation and cell expansion during nectar pouch development. Distances between every pair of landmarks recapitulate shape information as much as landmark coordinates **(A)**. The calculation of proliferation rate and cell expansion contributions to landmark inter-distance dynamics is presented **(B)**. Three triplets of petals corresponding to early, mid, and late stages and one adult petal have been used to study the contributions. For each individual, pairs of landmarks that are left-right symmetric have been averaged, and the measures of each triplet have also been averaged. The relative contributions of cell proliferation (light blue) and expansion (pink) are summarized in **(C)**. For each comparison (early to mid, mid to late, and early to late), landmarks have been ordered according to increasing inter-distance values between the two stages. Green bars correspond to the average rate of the increase in inter-landmark distance between consecutive stages.

Geometric morphometrics is based on the coordinates of landmarks optimally aligned through a series of steps, one of which removing the effect of scale. This implies that any shape variation can be viewed as a differential evolution of the distances between pairs of landmarks. Consequently, we used this assumption to investigate the number and size of cells that are located between every pair of landmarks corresponding, in dorsal view, to the nectar pouch (Figure 4A). We defined three stages (i.e., early, mid, and late plus adult stages; Figure 4B), and for each stage, we calculated cell number and absolute length between every pair of landmarks. Three individuals for each stage were used for averaging purposes, and a left/right average was also performed to match the geometric morphometric analyses, which use only the symmetric component. We then calculated what we called the cell proliferation contribution (*Cp*) and the cell expansion contribution (*Ce*) as follows:


Cpi,j(stage 2-stage 1)=(Ni,j(stage 2)-Ni,j(stage 1)Ni,j(stage 1))/(Di,j(stage 2)-Di,j(stage 1)Di,j(stage 1))×100

Cei,j(stage 2-stage 1)=100-Cpi,j(stage 2-stage 1)

where *N^i,j^* is the cell number between landmarks *i* and *j* at a given stage, and *D^i,j^* is the distance between landmarks *i* and *j* at a given stage, which measure, for a given pair of landmarks and between two stages, what percentage of the increase in absolute distance between these two landmarks can be attributed to cell proliferation or expansion, respectively (Figures 4B,C). More precisely, *Cp* is equal to proliferation rate (i.e., the number of supplementary cells compared with the initial amount, in percentage) divided by the expansion rate (i.e., the supplementary length compared with the initial distance, in percentage). *Ce* is equal to 100−*Cp*. We did not observe any decrease in the length or number of cells between two landmarks, from a young stage to an older stage, resulting in exclusively positive values for *Cp* and *Ce*.

Some landmark pairs target regions that contribute more than others to the shape variation during development, but the identification of these regions can be obscured by the Procrustes fit. We, therefore, used an alternative way to classify their contributions by attributing a “growth score” to each pair of landmarks, i.e., calculated as the ratio of the distance between them at the oldest stage divided by the length at the youngest stage.

## Results

### Geometric Morphometrics of the Developing *Nigella damascena* Petal: An Allometric Law Governs the Covariation of Petal Shape and Size Dynamics During Development

To depict the allometric patterns of covariation between shape and size, we used a bivariate representation of their proxies (i.e., the first principal component of shape variation and centroid size, respectively). For the dorsal view, shape follows a fairly clear developmental path from the youngest to the oldest stages (Figure 2A). The variation of shape of the ventral view globally shows a pattern similar to that of the dorsal view, with the exception that late stages exhibit a bifurcation along the first axis (Figure 2B). This is explained by the positioning of the upper lip in either an open or closed position (the second component shows a trend closer to the dorsal one). For the medial view, we observed a similar trend for early to intermediate stages, as later stages show a plateau in terms of shape variation (Figure 2C). For the lateral view, the first two principal components of shape capture less shape variation (40 and 30%) than for the other views, and no clear developmental trajectory emerges from their pattern of covariation with size.

Covariation between size and global shape variations is well approximated by a mathematical relation at least for dorsal view, which, at a first approximation, seems exponential as it is linearized by applying log to size (Figure 3B). In other words, during overall petal development, dorsal shape (but also ventral and sagittal, although to a lesser degree) shows a shape acquisition exponentially slower than size. The morphometric analyses of the subsets of landmarks, corresponding to the stalk (Figure 3C, 4 landmarks) or the nectar pouch (Figure 3D, 6 landmarks), compared with overall petal analysis (Figure 3A, 10 landmarks), suggest that each substructure of the organ seems to behave similarly to the overall petal (in the sense that, to a given increase of size, the corresponding increment of shape change is lower and lower as the size increase). In particular, applying log to size partially linearizes the covariation between shape and size for only either stalk or nectar pouch (Figures 3C,D, respectively); however, it is much less clear than for the entire petal. The real relationship between size and shape is probably more subtle than a simple exponential law.

### Disparities in Cell Characteristics on the Dorsal Part of the Nectar Pouch During Development

**FIGURE 5:**
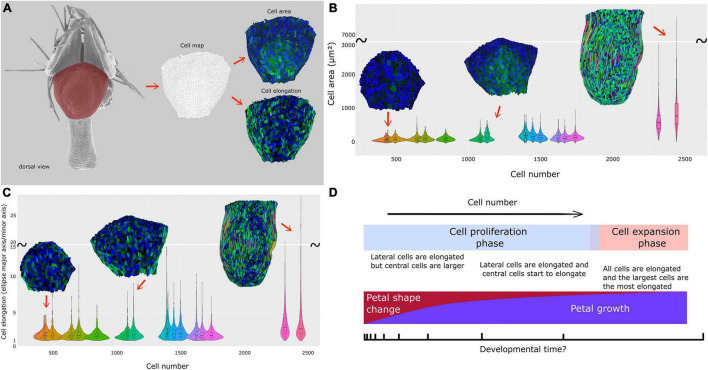
Cell dynamics during petal morphogenesis with focus on a dorsal view of the nectar pouch. **(A)** The petal dorsal view of the nectar pouch has been transformed into cell maps to measure and visualize cell area (up) and cell expansion (down) dynamics. Color code is detailed in Supplementary Figure 1. Dynamics and distribution of cell area **(B)** and elongation (major axis/minor axis of the equivalent ellipse) **(C)** are visualized through violin plots, themselves plotted against total cell number. **(D)** Summary of macroscopic and cellular characteristics of *N. damascena* petal during development.

The dorsal part of the nectar pouch can be clearly delimited from the neighboring domains (i.e., lobes and stalk) from early to late stages, and as seen earlier, its shape acquisition follows a clear developmental trend. The relationship between its shape and size follows the same allometric relation as the overall petal. Together, these characteristics make the dorsal part of the nectar pouch a key model to study cellular processes (Figure 5A).

Cell number and mean cell surface were computed on the dorsal views of the nectar pouch. Despite a lack of resolution for older stages, the mean cell surface is globally constant, around 20 μm^2^, from early to late stages, when suddenly mean cell size strongly increases to reach about 80 μm^2^ (Supplementary Figure 3A). In parallel, in the first phase, cell number increases proportionally faster than petal size, but then it increases much slower when cells undergo cell growth (Supplementary Figure 3B).

The cell maps drawn from the dorsal views of the nectar pouch during development allowed us to highlight the diversity of cell behaviors in terms of shape and size changes, depending on their localization on the tissue. In the earliest stages, all cells have approximately the same size, but variance progressively tends to increase, and while the largest cells are located in the middle of the pouch, the most elongated ones are distributed on the lateral sides (Figures 5B,C and Supplementary Figure 1). Finally, as the pouch grows, the largest cells are also the most elongated ones, suggesting anisotropic expansion. Most of them are localized on the lateral sides, but central cells are also affected by elongation. Cells at the boundary between lobes and nectar pouch (i.e., the topmost cells in these maps) are characterized by small size and relatively limited elongation, even in the latest stages.

### Disentangling the Contribution of Cell Proliferation and Cell Expansion in the Acquisition of the Final Shape and Size of the Nectar Pouch

If cell proliferation and cell expansion are the two major cell events that influence petal shape acquisition and growth, their respective contributions may vary during the petal development and remain to be determined.

The pairs of landmarks are arranged by increasing growth scores, measured as the ratio of the distances between consecutive stages (Figure 4C). Pairs whose score is higher or lower than the average can be considered as the ones contributing the most to the evolution of the overall shape. This permits to highlight a great difference in cell proliferation and growth contributions depending on the pairs of landmarks. In particular, it is interesting to note that between the early and intermediate stages, which is the most important interval in terms of shape patterning, cell proliferation is predominant for landmark pairs that undergo the largest expansion (with a high growth score, located at the right of Figure 4C) and almost absent for landmark pairs that undergo the smallest expansion [with a low growth score, located at the left of the graph (Figure 4C)]. Between intermediate and late stages, cell expansion becomes predominant (>80% for all pairs of landmarks).

## Discussion

Complex petal shape elaborations have been qualitatively described in many taxa. They are very common and diverse in some of them, such as Ranunculaceae or Rosids, and rarer in other taxa such as Asterids (Endress and Matthews, 2006). How these elaborations are acquired during development can also be found from numerous developmental studies (Kosuge and Tamura, 1988; Erbar et al., 1999; Tucker and Hodges, 2005; Sauret-Güeto et al., 2013; Sharma et al., 2014). However, the covariation of shape and size entailed by the progressive acquisition of the various elaborations contributing to a complex shape has been rarely studied. This study is the first attempt at this characterization using the Ranunculaceae species *N. damascena* and combining the analyses of geometric morphometrics and cell characteristics. We found that shape changes more rapidly than size increases at early stages, marking a nonlinear allometric relationship between them. In other words, the different domains constituting the mature petal differentiate quite early during development, well before the final size is reached. We also found that cell proliferation plays a major role in shape transformation, contrary to cell expansion, which rather contributes to petal growth. This strongly suggests that morphogenetic cues, whatever their nature, preferentially act through the spatial and temporal control of cell proliferation, while petal size establishment is controlled by cell expansion.

### Developmental Patterns of Petal Shape and Size Dynamics

Size increases as development proceeds. To capture the complex shape of petals across their developmental range of size variation, each of the four views (i.e., dorsal, ventral, medial, and lateral) allows a focus on specific regions and transects, which give information not available in the other views (e.g., upper lip for ventral view or the bottom of the nectar pouch for medial view). At the exception of most of the shape of the lobes (including the pseudonectaries), every petal domain has been documented in this analysis, which was important considering that all these domains, as well as, in particular, their shape and arrangements, contribute in one way or another to the final shape of the petal as a biologically functional unit. For example, due to the location of the nectar at the bottom of the pouch, the stalk (and, in particular, its length) defines the position of the insect when it feeds. In the same way, nectar pouch size and shapes are crucial for nectar storage and reward access.

For a given view, we might have missed petal shape information because (i) SEM pictures are the 2D projections of a 3D shape, and (ii) the number of landmarks summarizing the overall shape is limited. These limitations impact the multidimensional analysis of shape. Nevertheless, several clues suggest that the PCA first axes are good proxies to follow petal shape variation during development, as long as the real ontogenetic trajectory is linear. First, 80% of the variation is carried by the first axis for dorsal and ventral views. Second, plots against centroid size show a clear developmental trend for three of the four views (i.e., distributions are sparsely dispersed). Among the four views, the dorsal view presents the clearest trend. The ventral view exhibits a bifurcation in large petals, explained by the presence of the upper lip, which is long enough in late stages to be either in an opened or closed position according to the sample, hence affecting the landmark positions and so the global shape. Many factors, such as specimen preparation, could explain why the upper lip is in one or the other position for a given petal. However, if it strongly affects the global shape analysis, it is less relevant from a biological perspective. Nevertheless, the second principal component, while capturing only 10% of the total variation, does not discriminate the petals according to this “opening” criterion and presents a distribution close to that of the first component for the dorsal view. The sagittal medial view shows a clear trend for the young-to-medium individuals and then probably exhibits a plateau, although studying additional mature specimens would be needed to confirm it. Nevertheless, as this plateau is also observed for the second and third axes, it suggests that the final sagittal shape is reached earlier than the final size. Finally, the lateral view does not show any clear developmental trend (i.e., shape variation between two different developmental stages was not higher than that between two organs at the same stage), probably due to the lack of a sufficient sample of the early-stage petals and a very-large inter-individual variation.

### Origin of the Allometric Relation, Which Links Petal Pouch Shape and Size Dynamics

The first axis of PCA coordinates variation, and the relative distance between two individuals on the axis can be interpreted not only qualitatively but also quantitatively and therefore constitutes a proxy for studying the dynamics of the variation in petal shape.

In this study and particularly for the dorsal view, the allometric relation observed between the dynamics of shape and the dynamics of size seems to be fairly approximated by an exponential law. Similar trends are also found for subdomains such as stalk or nectar pouch. To exclude an effect due to the unequal sampling of different stages in terms of size distribution, the Procrustes analysis and the PCAs were repeated using a subsampling procedure (Supplementary Figure 2). For all subsamplings, an exponential law is found again, which leads it to a more robust biological interpretation.

Nevertheless, more replicates would be needed to distinguish from an exponential law to a power law or another relationship (Figures 3E,F).

Several scenarios can explain this relationship between shape and size dynamics. In particular, such tight covariation may imply that there is a control of one parameter over the other, which in turn causes this quantitative relationship between them. On the contrary, as correlation is not causation, petal shape and size may not be linked causally but to a third parameter (e.g., developmental time), which could also cause such covariation.

In fact, the cell counts we performed suggest this last scenario. The relationship between cell number and petal surface seems globally linear in the early-to-mid stages (Supplementary Figure 3B), or, equivalently, the average surface of a cell is globally constant (around 20 μm^2^, Supplementary Figure 3A) during the first part of the development.

If cell proliferation rate is assumed to be constant through developmental time (at least during this first part of development), it entails that the dorsal part of the petal nectar pouch follows an exponential growth. Consequently, every biological process that has a constant dynamic through developmental time will be automatically linked to the size of the dorsal part of the petal nectar pouch exponentially, even if there is no biological link between them. We assumed that it may be the case for shape changes and that the allometric relationship between shape and size is not causal for *N. damascena* petal nectar pouch. Although all of these assumptions concerned the early-mid “proliferation phase,” which is also the phase where the shape changes the most, the “expansion” phase that follows may accentuate the shape of the distribution by stretching it at its tail. This probably explains why the distribution of shape against size does not clearly appear under two distinct phases, yet observed and measured on cellular parameters.

A complete and definitive confirmation of these assumptions (summarized in Figure 5D) would, however, require additional studies, such as real-time tracking of cell division during petal development.

### Differential Contributions to Petal Size and Shape of Both Cell Division and Cell Expansion

External tissue shape derives from the four primary characteristics of cell features (Guillot and Lecuit, 2013): not only cell number and size but also cell shape and arrangements. These characteristics originate from growth processes, mainly cell proliferation and expansion, and from their local variation in rates and orientation (Rolland-Lagan et al., 2003). The distances between all pairs of landmarks carry equivalent information to shape coordinates and, therefore, recapitulates the shape, as coordinates do. This is the reason why we chose to measure the number and size of cells along transects between every pair of landmarks and compare them among the developmental stages. Contrary to many animal tissues, cell rearrangements (and cell death) are rare in plant epidermis (Dickman et al., 2017), but if they happened, they would be indistinguishable from cells produced by mitosis, and the real-time tracking of cell division would be needed to distinguish between the two phenomena. Nevertheless, if a cell moved and got inserted into the transect between two landmarks, this event would be considered as cell proliferation as it would affect the number of cells. Alternatively, cell shape variation affecting cells between two landmarks would be considered as cell expansion events (including cell size decrease) as they can affect cell width on this line. The four primary characteristics could hence be considered as two main cell characteristics, which are easily measurable between two landmarks. On the one hand, counting the number of cells between two landmarks and comparing this number between two stages will give access to a mean value of proliferation. Dividing the absolute distance between the two landmarks by the number of cells gives, on the other hand, the mean cell size and so the mean value of cell expansion through comparison between two stages.

Even if cell number and cell size can be characterized, it is not trivial to deduce their relative contributions to shape dynamics because the shape is reflected by the relative distances between landmarks and not directly the distances themselves. Even if the Procrustes analysis does not give access to this information, we managed to build some bridges between cell characteristics (i.e., microscopic level) and shape changes as reconstructed by geometric morphometrics (i.e., macroscopic level). We considered that pairs of landmarks that undergo extreme variations (i.e., the farthest from the mean) in terms of the relative distance between two stages are the ones that participate the most to shape variation. Thereby, we found that the lengths of the external ridges of the pouch (1–2, with its symmetric 5–6, Figure 4C) and, at the other extreme, the between lobe sinus width (3–4) are the major contributors to shape dynamics. Pairs of landmarks concerning petal length tend to undergo higher increases than those concerning petal width. This is coherent with the major axis of cell elongation visualized on cellular maps (Figure 5 and Supplementary Figure 1).

The comparison between early-mid and mid-late graphs shows that cell proliferation has a preponderant role in the youngest stages, especially for the pouch width and overall length, which coincides with the phase where the shape changes the most. In later phases, cell expansion takes over, which supports the development in two phases, namely, first mainly proliferation and then mainly expansion, as has been reported for many plant organs (Rolland-Lagan et al., 2003; Puzey et al., 2012; Walcher-Chevillet and Kramer, 2016).

Finally, in the early-mid graph, corresponding to the crucial period in terms of shape changes, the pairs of landmarks that know the highest differential growths are governed at 50% or more by cell proliferation. On the contrary, in the pairs of landmarks that know the lowest differential growths (i.e., 3–4 and 1–6 pairs, corresponding between lobe sinus width and stalk width), cell proliferation is absent or almost absent, contrary to cell expansion. As a result, cell proliferation seems to be the predominant factor in shape dynamics and could therefore be the preferential cellular process controlled by the morphogenetic actors.

### What Are the Potential Contributions of This Study to the Understanding of Petal Diversity in *Nigella* and Other Genera of Ranunculaceae?

Petal shape and elaborations are quite diverse in the genus *Nigella* (Yao et al., 2019). The authors have focused on the timing of elaborations during development in a comparative framework. They have shown that most cellular elaborations and species-specific elaborations such as upper lip tail, ridges, pseudonectaries, begin to appear at the S7 stage, which is comparable to our mid-stage. If the bi-phasic development of the petal shape that we observed in *N. damascena* is conserved among species, this means that elaborations take place at different stages at the end of the proliferation phase and during the expansion phase. In contrast, the overall outline of the petal shape could be acquired during the first developmental phase governed by cell proliferation, with species-specific local differential growth rates. Expanding our approach to species with contrasting shapes such as *Nigella integrifolia*, *Nigella orientalis*, and *Nigella arvensis*, for example, would help to understand the developmental bases of shape diversification in the genus.

One of the elaborations of the *Nigella* petal is the nectar pouch. While we did not directly examine the contributions of cell proliferation and cell expansion to the deepening of the pouch, the cell maps and the growth scores of the distances between landmarks (e.g., 1–5/2–6, 1–4/3–6) support also a bi-phasic developmental process. In Ranunculaceae, spurred petals are encountered in the genus *Aquilegia* and in the tribe Delphinieae. In *Aquilegia*, the spur is established through an early phase of localized cell proliferation, followed by a phase of anisotropic cell elongation. Interestingly, cell expansion and more specifically its duration account for 99% of the differences in spur elongation among species (Puzey et al., 2012). Because the ancestor of the Ranunculaceae is devoid of petal nectar spur, and *Aquilegia* and *Nigella* belong to different subfamilies (Cossard et al., 2016; Zhai et al., 2019), the spur in *Aquilegia* and the pouch in *Nigella* cannot be considered as homologous structures. However, hollow petals appear to originate according to similar two-phase developmental processes. The Delphinieae tribe that encompasses all other Ranunculaceae species with spurred petals is the sister group of Nigelleae, raising the possibility of homology between the pouch and the spurs in these tribes. The hollow part of the petal spurs in these species has very diverse shapes (Zalko et al., 2021), including different lengths, and it is much deeper than the *Nigella* petal pouch. From an evolutionary perspective, it would be interesting to compare the dynamics of spur and pouch development in Delphinieae species and in *Nigella*, to establish whether a bi-phasic developmental process is also at play in Delphinieae and, if so, if the spur/pouch morphological differences could be accounted for by differences in the duration of cell proliferation and/or expansion phases.

## Data Availability Statement

The raw data supporting the conclusions of this article will be made available by the authors, without undue reservation.

## Author Contributions

PG, FJ, and CD designed the experiments. ML, FJ, and CD grew, collected, and sampled the material. PG designed and performed the morphometric analyses and wrote the manuscript. SG contributed to the morphometric analyses. PG and CD designed and performed the cell map analyses. All authors contributed to the article and approved the submitted version.

## Conflict of Interest

The authors declare that the research was conducted in the absence of any commercial or financial relationships that could be construed as a potential conflict of interest.

## Publisher’s Note

All claims expressed in this article are solely those of the authors and do not necessarily represent those of their affiliated organizations, or those of the publisher, the editors and the reviewers. Any product that may be evaluated in this article, or claim that may be made by its manufacturer, is not guaranteed or endorsed by the publisher.

## Supplementary Material

Supplementary Figure 1 The sequence of cell maps colored by cell area or cell elongation values. Cell maps for cell area **(A)** and cell elongation **(B)** are ordered from top left to bottom right in increasing total surface. For every individual, a common color code has been used to make easier comparisons between stages.

Supplementary Figure 2 Resamplings confirm the presence of an allometric law between shape and size during nectar pouch development. PC1 plots against centroid size **(A,C,E)** or log centroid size **(B,D,F)** after original sampling (characterized by an oversampling of smallest petals), equilibrate resampling (i.e., individuals regularly spaced on the size scale), and oversampling of largest petals. An exponential relation is found for every case, excluding a sampling bias and consolidating its biological origin.

Supplementary Figure 3 Cell characteristics (mean size and number) during development. **(A)** Cell number plotted against nectar pouch surface (mm^2^). **(B)** Mean cell size (μm^2^) plotted against cell number shows a stasis phase of mean cell size (compatible with a proliferation phase), which ends with a sharp increase in cell mean size (compatible with an expansion phase).
