# Supplementary figures and images for: Micro- and Macroscale Patterns of Petal Morphogenesis in Nigella damascena (Ranunculaceae) Revealed by Geometric Morphometrics and Cellular Analyses
Source: Front Plant Sci. 2021 Nov 19;12:769246. doi: 10.3389/fpls.2021.769246 (PMC8640125; doi:10.3389/fpls.2021.769246)

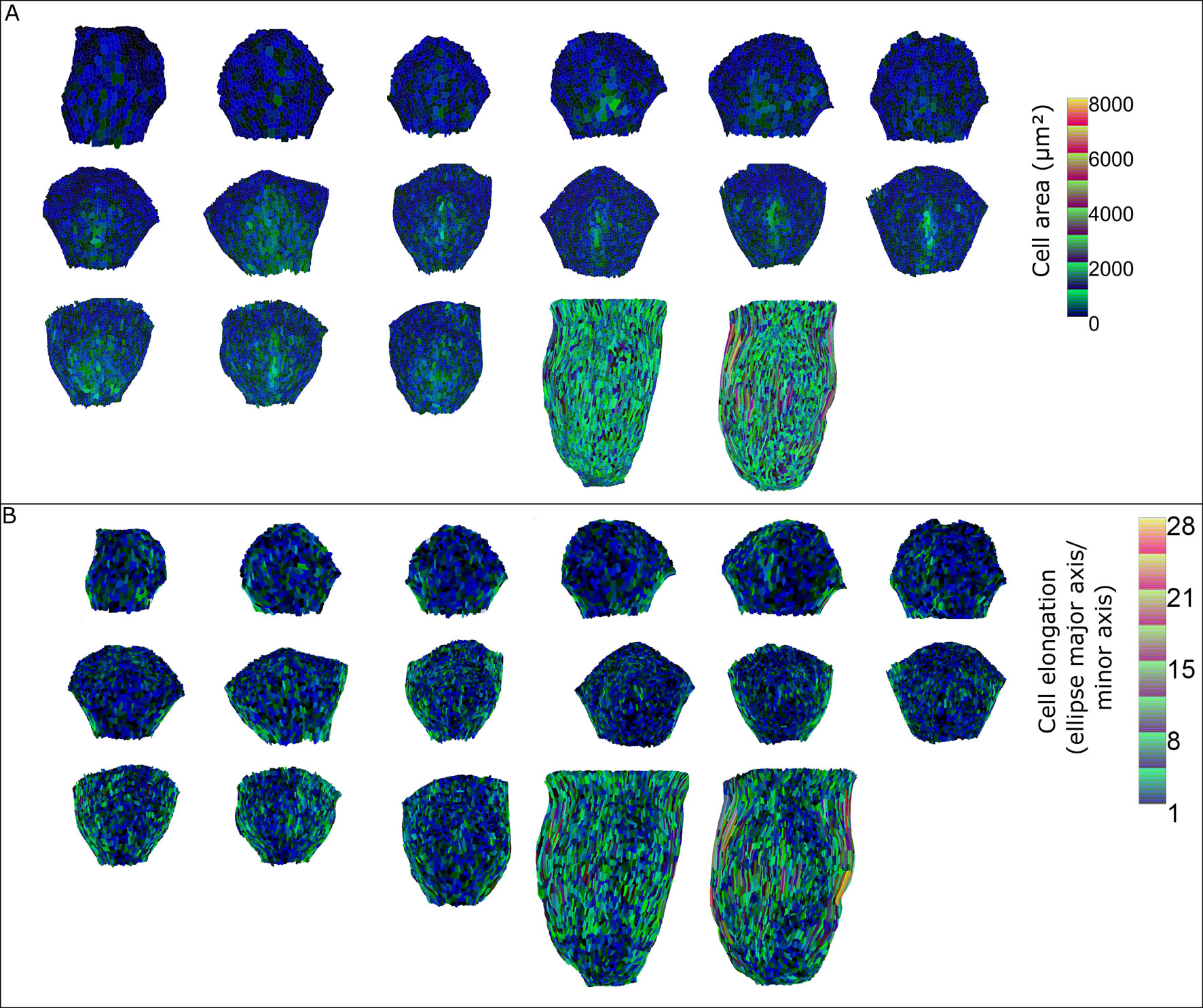

Supplement: Supplementary Figure 1 — The sequence of cell maps colored by cell area or cell elongation values. Cell maps for cell area (A) and cell elongation (B) are ordered from top left to bottom right in increasing total surface. For every individual, a common color code has been used to make easier comparisons between stages. [file Image_1.jpeg]

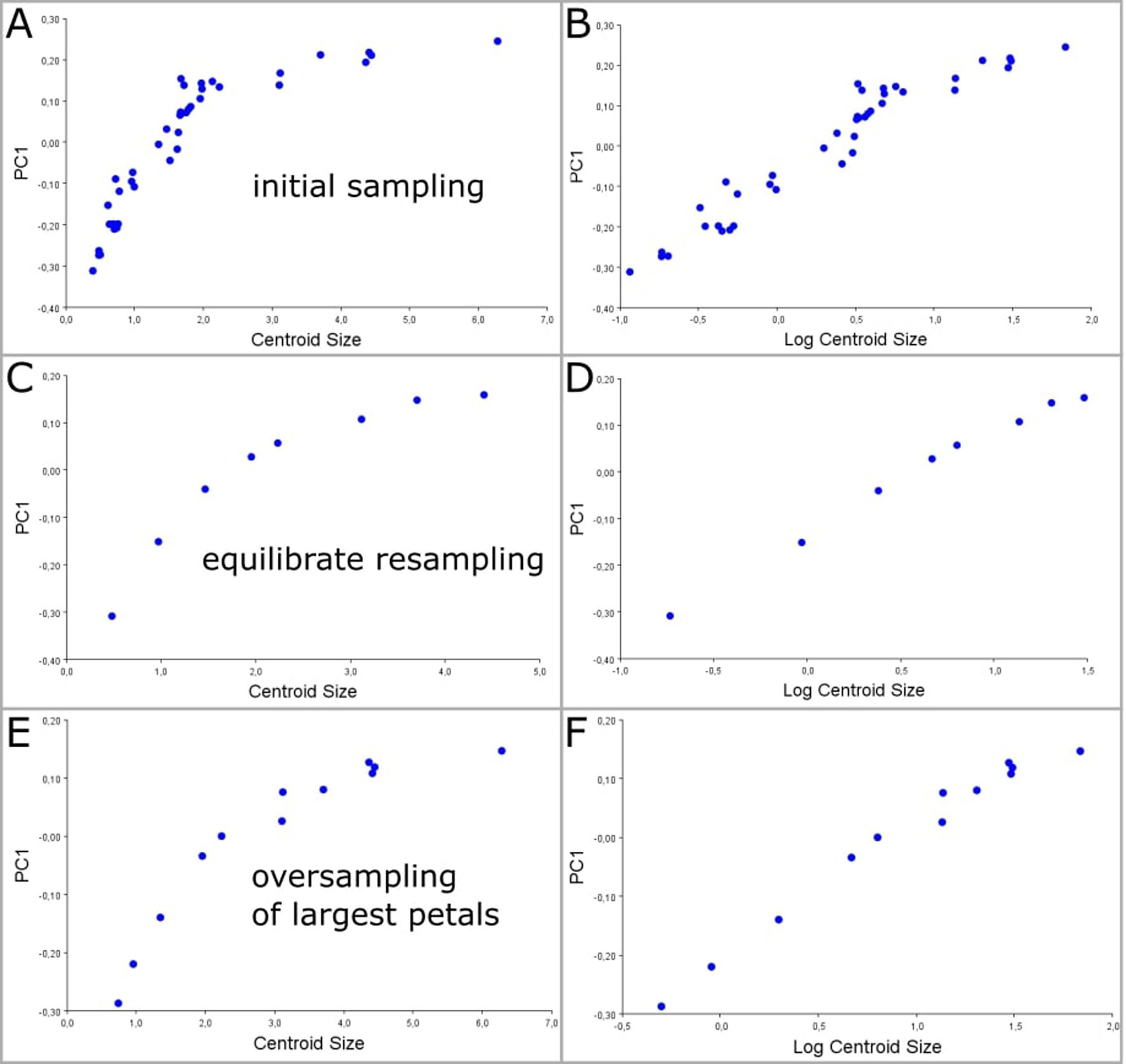

Supplement: Supplementary Figure 2 — Resamplings confirm the presence of an allometric law between shape and size during nectar pouch development. PC1 plots against centroid size (A,C,E) or log centroid size (B,D,F) after original sampling (characterized by an oversampling of smallest petals), equilibrate resampling (i.e., individuals regularly spaced on the size scale), and oversampling of largest petals. An exponential relation is found for every case, excluding a sampling bias and consolidating its biological origin. [file Image_2.jpeg]

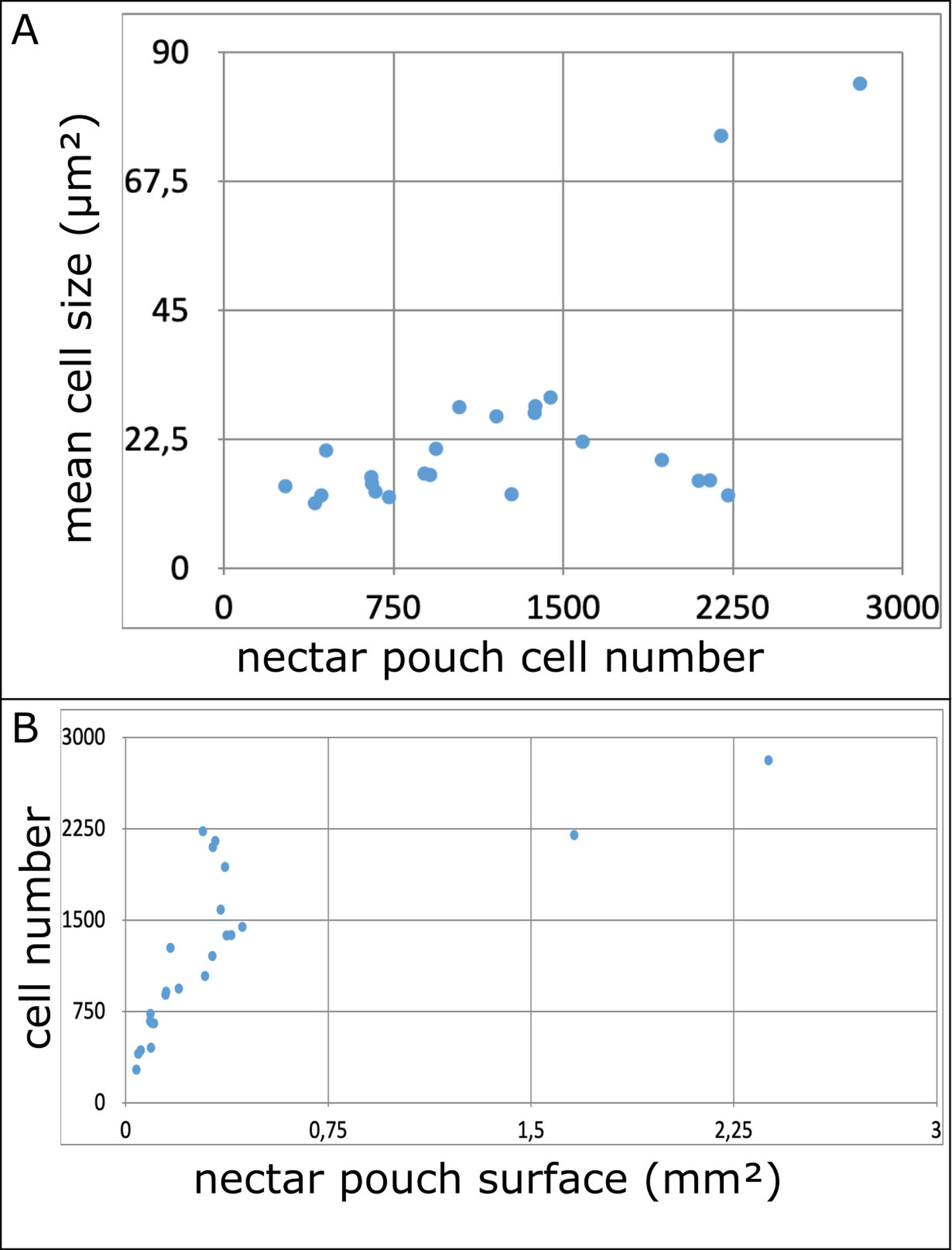

Supplement: Supplementary Figure 3 — Cell characteristics (mean size and number) during development. (A) Cell number plotted against nectar pouch surface (mm2). (B) Mean cell size (μm2) plotted against cell number shows a stasis phase of mean cell size (compatible with a proliferation phase), which ends with a sharp increase in cell mean size (compatible with an expansion phase). [file Image_3.JPEG]
